# Theoretical modeling and neritic monitoring of loggerhead *Caretta caretta* [Linnaeus, 1758] sea turtle sex ratio in the southeast United States do not substantiate fears of a male‐limited population

**DOI:** 10.1111/gcb.15808

**Published:** 2021-07-28

**Authors:** Michael D. Arendt, Jeffrey A. Schwenter, David W. Owens, Roldán A. Valverde

**Affiliations:** ^1^ Marine Resources Division South Carolina Department of Natural Resources Charleston SC USA; ^2^ Grice Marine Laboratory College of Charleston Charleston SC USA; ^3^ Department of Biological Sciences Southeastern Louisiana University Hammond LA USA; ^4^ Sea Turtle Conservancy Gainesville FL USA

**Keywords:** Atlantic Multidecadal Oscillation, climate, demography, sea turtle, sex ratio

## Abstract

Sea turtles are among several hundred species whose sex is determined by incubation conditions during critical developmental periods. Consequently, these marine reptiles may be vulnerable to global climate change, and under the assumption of continued climate warming, numerous studies pose dire predictions for future populations based primarily on hatchling sex ratio data. Alternatively, as long‐lived species that take decades to reach maturity, without inherent coping mechanisms for such change, sea turtles could not have persisted across geological epochs. Globally, loggerhead *Caretta caretta* [Linnaeus, 1758] sea turtles occupy temperate zones, with ontogenetic development that spans the entirety of gyres associated with respective ocean basins. The largest rookery for this species occurs in the Northwest Atlantic Ocean (NWA) population, where a 30‐year cycle in annual nest counts is reported through 2018. Complementary studies document a lagged association between these annual nest counts and the Atlantic Multidecadal Oscillation (AMO); however, the underlying mechanism for this association remains elusive. Therefore, objective 1 evaluated the effect of AMO‐mediated cohort resonance on the demographic structure of a theoretical neritic assemblage under variable cohort abundance and female proportion but stable annual survival during 165‐year runs (i.e., extent of AMO data). For objective 2, blood samples were used to assign sex to 2217 loggerhead sea turtles captured by research trawling (2000 to 2019) on the inner continental shelf from St. Augustine, FL (29.9°N) to Winyah Bay, SC (33.1°N). Shorter oceanic duration of less female‐biased cohorts from the AMO cold phase synchronized peak adult male and adult female co‐occurrence during subsequent warm phases three decades later. Grand sex ratio predicted from testosterone was 67% female (*n* = 1484), with a slight temporal female decline. Our findings suggest greater population sex ratio plasticity than predicted solely from terrestrial nesting data.

## Graphical abstract

Shorter oceanic duration of cohorts with 75% versus 95% female bias during AMO cold phases synchronized peak adult female (first *y*‐axis) and adult male (second *y*‐axis) co‐occurrence in subsequent AMO warm phases. A 15‐year smooth oscillation in hatchling abundance (model M5) influenced adult female abundance, but not the ratio of adult males to adult females. These findings, created with fixed stage‐based annual survival, highlight the ecological contributions of climate in governing population structures, as well as offers novel insight regarding a potential evolutionary advantage of environmentally mediated sex determination for long‐lived, late‐maturing species.

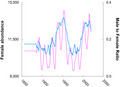

## 1 INTRODUCTION

Sex ratio is one of the most important demographic factors for the long‐term viability of interbreeding groups of organisms; a minimum number of females is needed for population abundance, while a minimum number of males ensures adequate genetic diversity and fertility. For genetic sex determination (GSD), Fisher (1958) noted that males and females are not birthed equally, but rather that these differences are offset by sex‐linked rates of reproduction and death. For environmentally mediated sex determination (ESD), Charnov and Bull (1989) contended that population sex ratio reflects differential fertilities (a relative measure of fitness) of males vs. females. Furthermore, Charnov and Bull (1989) concluded that the sex with lower mean fertility is “overproduced” to compensate for future environmental uncertainty and, thus, is akin to a limiting reagent in a chemical reaction. Overproduction of the least fit sex therefore increases the probability that a suitable number of individuals of this sex will exist at maturity to maintain a population, which is particularly important for long‐lived and late‐maturing species.

Loggerhead *Caretta caretta* [Linnaeus 1758] sea turtles are long‐lived and late‐maturing reptiles, whose sex is determined by the proportion of development at temperature equivalents during the middle third of incubation (Georges, 1989; Standora & Spotila, 1985; Wibbels, 2003). The transition between strong male (long, cool incubation periods) versus female‐biased clutches spans just a few degrees, with pivotal temperatures that produce equal numbers of each sex around 28–30℃ for most sea turtle species (Wibbels, 2003). Although nest chambers are located ~45 cm deep (Hanson et al., 1998), only the peripheral eggs interface with sand; thus, embryonic development occurs in a “physiologically dynamic” substrate that more closely resembles “water or agar” than sediment (Wyneken & Lolavar, 2015). In this regard, neither soil nor air temperatures alone adequately reflect the “microclimate” and “microhabitat” experienced by developing eggs (Wyneken & Lolavar, 2015). Consequently, with sufficient intra‐daily temperature variation, all female clutches can be produced at temperatures <28ºC (Georges et al., 1994). Likewise, with sufficient moisture content, all male clutches can be produced at incubation chamber temperatures >30ºC (Wyneken & Lolavar, 2015).

In the northwest Atlantic Ocean (NWA), the epicenter of loggerhead sea turtle nesting occurs in central to southeast Florida (Witherington et al., 2009), where 85%–90% of hatchlings are female (Mrosovsky & Provancha, 1992; Wyneken & Lolavar, 2015). Higher latitude beaches are associated with considerably less female bias (Mrosovsky, 1988) and greater within‐season sex variability (Mrosovsky et al., 1984), but contribute <10% to regional nesting (NMFS & USFWS, 2008). Sex ratio variability in loggerhead sea turtles at lower latitudes is predominantly driven by precipitation, but annual reductions in the female proportion of clutches do not appear to occur frequently enough (Wyneken & Lolavar, 2015) to deviate considerably from the assumption of ~90% female (Mrosovsky & Provancha, 1992). Consequently, ≥85% of annual sea turtle hatchlings entering the NWA annually are likely female. As such, predicted increases in atmospheric warming beyond unrivaled rates of temperature change relative to the past 800 years (IPCC, 2013) are worrisome for the sex ratio structure of future populations for this and other species with ESD.

Although more than 400 species exhibit ESD (Lockley & Eizaguirre, 2021), sea turtles have received the most attention with >250 publications on the topic of climate threats to population sex ratio published since 1988 (Hawkes et al., 2009; Patrício et al., 2021). The direst prediction for future sea turtle populations is an insufficient number of males for breeding and subsequent genetic diversity (Fuentes et al., 2010; Hawkes et al., 2007; Jensen et al., 2018). Reduced egg and hatchling survival and fitness at higher temperatures (Fisher et al., 2014; Laloë et al., 2017; Pike, 2014; Yntema & Mrosovsky, 1982) are also suggested, but which should theoretically decrease the relative female proportion. Under extreme scenarios, maternal mitigation measures such as nest site selection (Mortimer, 1990; Wood et al., 2000) and earlier nesting season onset (Weishampel et al., 2004) could prove inadequate for temperature compensation, particularly if suitable nesting habitat is unavailable due to sea level rise and shoreline armoring (Fish et al., 2005; Fuentes et al., 2011).

In stark contrast to nesting beach concerns, loggerhead sea turtle sex ratios on foraging grounds in the southeast United States appear to have remained remarkably similar between the early 1980s (Wibbels et al., 1991) and the 2000s (Arendt, Boynton, Schwenter, Segars, Byrd, Whitaker, Parker, Owens, et al., 2012) which is curious given considerable corresponding changes in atmospheric temperature (IPCC, 2013). In the NWA, hatchling loggerhead sea turtles rapidly recruit to pelagic habitats near the edge of the continental shelf (Witherington, 2002), then shift into the oceanic realm and remain for 7 to 12 years before returning to the continental shelf (Bjorndal et al., 2000). Variable neritic recruitment (NR) age, particularly if environmentally mediated, could amplify cohort resonance and in turn influence neritic assemblage demographics (Bjørnstad et al., 2004). Therefore, objective 1 modeled sex ratio for a theoretical neritic assemblage, where NR age and cohort sex ratio varied as linear functions of the Atlantic Multidecadal Oscillation (AMO) which has a lag association with loggerhead sea turtle nesting in Florida but for still uncertain reasons (Arendt et al., 2013; Van Houtan & Halley, 2011). For objective 2, we updated and evaluated sex ratio data from an important neritic sea turtle foraging ground in the southeast United States (Arendt, Boynton, Schwenter, Segars, Byrd, Whitaker, Parker, Owens, et al., 2012) in the context of model outputs.

## 2 MATERIALS AND METHODS

### 2.1 Theoretical modeling

A matrix model containing five life‐history stages was constructed to evaluate changes in the demographic structure (i.e., age by sex) of a theoretical neritic assemblage. Annual hatchling cohorts (H) represented the first life‐history stage (a) in the matrix model, with annual abundance denoting post‐ocean entry such that hatchling abundance reflected cumulative variability in annual nest counts, clutch size, and successful hatching and reaching the sea. Subsequent stages consisted of (b) age 0 survivors (A0S) to distinguish between entering the ocean and locating suitable protective habitat (Witherington, 2002); (c) annual oceanic survival (OS) for a minimum of 7 years (Avens et al., 2013); (d) variable NR age for initial departure from the oceanic realm and subsequent neritic recruitment; and (e) continued annual neritic survival (NS) to age 77, a minimum estimate of maximum NWA loggerhead sea turtle age per Avens et al. (2015).

The matrix model was built across worksheets contained within a single Microsoft (MS) Excel (Microsoft Corporation, Version 2016) file. The first sheet captured annual A0S, OS, and NS, with first application of NS in the year of NR. Given emphasis on climate‐mediated hatchling sex on long‐term population structure, annual survival remained fixed for A0S (0.4), OS (0.713), and NS (0.854), but survival variability is explored elsewhere (Arendt, M., & Schwenter, J., in review). Survival values were obtained or extrapolated from Table 1 for a stable (vs. exponentially growing) NWA population per the 2009 Global Status Review (Conant et al., 2009) which also indicated a high degree of parameter similarity across populations. Stage‐based survival rates were diagonally aligned for 242 cohorts, the first 77 of which “burnt in” the full complement of 78 cohorts (i.e., age 0 to age 77). Annualized cohort persistence was computed in a second sheet by diagonally populating A0S using the “offset” function and then routine multiplication thereafter through terminal cohort age (i.e., age 1 proportion = age 0 proportion × age 1 survival, etc.). In the third and final sheet, the proportion of each cohort remaining in year “*x*” was multiplied by initial abundance to generate an integer count of annual abundance per sex, computed separately. Consequently, the survivorship (*S*) equation uniformly applied to all 242 cohorts was:

$$S=\left[H\right]\times \left[A0S\right]\times \text{Power}\left(\left[\text{OS}\right],\;\left[\text{NR}\right]‐1\right)\times \text{Power}\left(\left[\text{NS}\right],\;77‐\left[\text{NR}\right]\right).$$

**TABLE 1:** Overview of annual hatchling abundance (H), corresponding sex ratio (SR), and cohort assigned age at neritic recruitment (NR). Dashes (—) indicate fixed annual parameter values

Model	H	SR	NR
M1	—	—	—
M2	—	—	AMO
M3	—	AMO (±5%)	AMO
M4	—	AMO (±10%)	AMO
M5	15‐year	AMO (±10%)	AMO

Five model structures were evaluated across combinations of variability in annual hatchling abundance, hatchling sex ratio, and NR age (Table 1). Combined sex annual hatchling abundance was fixed at *n* = 2,587,500 for models M1 to M4; annual hatchling abundance was the mean of 30k and 60k nests, the approximate 30‐year range reported by Ceriani et al. (2019), then multiplied by 115 eggs per nest (Conant et al., 2009) and presuming 50% entered the ocean. For model M5, annual hatchling abundance fluctuated along a 15‐year smooth oscillation to simulate the 30‐year trend reported by Ceriani et al. (2019). Annual (integer) nest counts were computed as “[SIN(Order/2.5) × 15,000] + 45,000,” where “order” (1 to 165) corresponded to 1856 to 2019. Annual hatchling (integer) abundance was then computed as described above for M1.

For models M1 and M2, cohort sex ratio remained fixed at 2,199,375 females (0.85) and 388,125 males (0.15) annually, but with lower female proportion in the AMO cool phase per historical soil temperature association for other models (Arendt, 2016). For model M3, annual female hatchling abundance spanned ±0.05 in concert with AMO‐mediated age as follows: 2,070,000 (8), 2,134,687 (9), 2,199,375 (10), 2,264,062 (11), and 2,328,750 (12). For model M4, annual female hatchling abundance spanned ±0.10 in concert with AMO‐mediated age as follows: 1,940,065 (8), 2,070,000 (9), 2,199,375 (10), 2,328,750 (11), and 2,458,125 (12). For models M3 and M4, annual male abundance was computed as 2,587,500 less the corresponding female abundance. For model M5, the proportion of annual female and male hatchlings reflected the NR‐derived ratio of model M4, with variable annual cohort abundance that generated a grand mean combined sex annual abundance of 2,613,185 (range = 1,725,000 to 3,449,942).

Annual NR for model M1 remained fixed at age 10 to ensure functionality but varied annually as a function of the AMO for models M2 to M5. Unsmoothed (standard, long format) monthly values were downloaded from the NOAA Earth Systems Research Laboratory; (http://www.esrl.noaa.gov/psd/data/timeseries/AMO/, accessed February 4, 2021) and parsed to April through November to reflect nesting and hatching season. Normalization of AMO data was computed as in the studies Van Houtan and Halley (2011) and Arendt et al. (2013): a grand mean between 1856 through 2020 was subtracted from each annual mean, and each corresponding annual value was divided by the grand standard deviation (SD), to produce a data series with a mean of 0 and a SD of 1. For each cohort year, series sums (age 0 through 7) determined NR age, with younger age reflecting published lag association with nesting: ≤19th percentile (NR = 8), 20th to 39th percentile (9), 40th to 59th (10), 60th to 79th (11), and ≥80th (12). For model simplicity, all members of a cohort recruited at the designated NR age/year.

### 2.2 Sea turtle capture, measurement, and blood collection

Loggerhead sea turtles were trawl‐captured (see Arendt, Boynton, Schwenter, Segars, Byrd, Whitaker, Parker, Owens, et al., 2012 for gear description) in coastal waters (5–14 m deep) between May and August between Winyah Bay, SC (33.1°N) and St. Augustine, FL (29.9°N). Trawling from 2000 to 2003, 2008 to 2015, and in 2018 and 2019 was conducted using a random sampling design (Arendt, Boynton, Schwenter, Segars, Byrd, Whitaker, Parker, 2012). In 2010, random trawling occurred in four subsets of geographic subregions (Arendt, Boynton, Schwenter, Segars, Byrd, Whitaker, Parker, 2012) to evaluate the impact of repeat trawling on recapture rates. Trawling in the Charleston, SC shipping channel was conducted in 2004–2007 (Arendt, Schwenter, et al., 2012) and again in 2016–2017, and is included herein to evaluate potential temporal change in sex ratio at this capture location. Target gear tow speed was 2.8 kts, with seafloor trawls towed for a maximum of 30 min except (a) between 2008 and 2010 when restricted to 20 min per Endangered Species Act, Section 10(a)(1)(A) permits issued by the National Marine Fisheries Service (NMFS), Office of Protected Resources (see Acknowledgements), and (b) in the shipping channel (Arendt, Schwenter, Segars, et al., 2012).

Multiple morphometric measurements were recorded (Bolten, 1999); however, herein we only report minimum straight‐line carapace length (SCLmin; cm) to estimate sea turtle age. Capture size was used to estimate age based on an equation generated from seven size and age coordinate pairs, corresponding to 5‐year intervals between ages 5 and 35, in figure 3c of Avens et al. (2013). Coordinate pairs were obtained using a screen grab image imported into MS Powerpoint and the corresponding guide tool. In MS Excel, coordinate pairs generated the equation “SCL = 18.705 × (age)^0.4403^” (*r* ^2^ = 0.98) which produced sizes of 44.1 cm SCLmin for age 7 and 110.6 cm SCLmin for age 77, comparable to small NR (Bjorndal et al., 2000) and large breeding adult sizes (Arendt, Segars, Byrd, Boynton, et al., 2012; Avens et al., 2013), respectively. To account for variability in somatic growth (Bjorndal et al., 2013), this size‐at‐age distribution was increased as well as decreased by 5% to bracket for impacts on age structure estimation.

### 2.3 Sea turtle sex prediction

Blood samples were collected from the dorsal cervical sinus of sea turtles using a 21‐ga, 1.5″ (3.8 cm) needle with hub and a 10‐ml heparinized vacutainer tube per Owens and Ruiz (1980). Vacutainer tubes were centrifuged for 5 min before plasma was pipetted into 2‐ml cryovials then frozen in a liquid N Dewar until transfer to a shore‐based −80℃ freezer. Plasma testosterone (T) concentration was measured using radioimmunoassay (RIA) described in detail by Blanvillain, Pease, et al. (2008). RIA is reliable at water temperatures >23℃ (Braun‐McNeill et al., 2007), and loggerhead sea turtles were captured at surface water temperatures of 26.9 ± 2.0℃ (mean ± standard deviation [SD]). Pre‐2004, loggerheads with T concentrations <200 pg ml^−1^ were identified as female, between 200 and 300 pg ml^−1^ as undetermined, and >300 pg ml^−1^ as male. Discontinuation of some of the original reagents for the assay in 2004 necessitated the adjustment of this scale after validation between new and old reagents. Since 2004, sex was assigned as female (<400 pg ml^−1^), undetermined (400 to 500 pg ml^−1^), and male (>500 pg ml^−1^); however, sex was cautiously assigned for loggerhead sea turtles ≥75.1 cm SCLmin, as pubescent females can be associated with elevated T concentrations (D.W. Owens, pers. obs.).

RIA was discontinued in 2019, and T concentrations were instead measured using an ELISA (enzyme‐linked immunosorbent assay; ENZO Life Sciences). T validation between RIA and ELISA is published for green (*Chelonia mydas*, Linnaeus) sea turtles (Allen et al., 2015) and has been completed but not published for loggerhead sea turtles (C.D. Allen, pers. comm. with JAS). An ELISA was performed using 50 µl for loggerhead (vs. ≤500 µl for RIA), with the same T concentration reference range as RIA since 2004 used to assign sex.

### 2.4 Statistical analyses

For objective 1, descriptive statistics across models characterized annual counts of males and females relative to three age groups: NR to age 19, 20–29, and 30–77. Age group delineations reflected estimated maturity at age 30 (Conant et al., 2009), with further distinction among presumed juveniles denoting expected duration in the neritic zone (Bjorndal et al., 2001) and the conservation value of protecting older juveniles (Crouse et al., 1987). Correlation testing (*α* = 0.05) performed in Minitab 18™ (Minitab Inc.) tested relatedness between annual female proportion and annual age group abundance across models. A second correlation test evaluated annual synchronicity between the abundance of age 30–77 females and the ratio of males to females in the corresponding age group across all five models.

For objective 2, Chi‐square analysis tested for differences in the assignment of loggerhead sea turtles to the three age groups above based on the somatic growth rate equation, with a fourth (expected) distribution created based on age structure distribution for model M1. Across the reconstructed, 5% slower, and 5% faster growth rate equations, size assignment to age groups were as follows: ≤19 (69.2, 65.7, 72.6 cm SCLmin); 20–29 (69.3 to 83.0, 65.8 to 78.9, 72.3 to 87.2 cm SCLmin); and ≥30 years old across growth rate equations. For each age group, the observed female proportion was evaluated for correlation with (a) sample size used to calculate the female proportion; (b) growth rate equation used to assign age, defined as 0 = −5%, 1 = reconstructed equation, and 2 = +5%; and (c) six chronological survey blocks as follows: 2000 to 2003; 2004 to 2007 (Charleston, SC shipping channel); 2008 to 2011; 2012 to 2015; 2016 and 2017 (Charleston, SC shipping channel); and 2018 and 2019 (terminal survey year). Pearson or Spearman correlation tests reflected continuous vs. factor data, respectively.

## 3 RESULTS

### 3.1 Objective 1

The base model (M1) consisted of 288,232 neritic loggerhead sea turtles, of which 80% were ≤19 years old, 16% were between ages 20 and 29, and the remaining 4% were between ages 30 and 77. Median age structure mirrored M1 when cohort NR age varied as a function of the AMO in models M2 to M4, but theoretical neritic abundance between 1856 and 2020 fluctuated between 164,740 (43% less than M1) and 545,406 (89% more than M1) individuals. Subsequently, loggerhead sea turtles ≤19 years old comprised 72% to 85% annually, with companion shifts between 12% and 22% for ages 20–29 and 3% and 6% for ages 30–77. Conversely, variable hatchling cohort abundance in model M5 produced theoretical neritic assemblages that ranged from 142,205 (14% less than M2 to M4) to 577,334 (6% more than M2 to M4) individuals. Likewise, loggerhead sea turtles ≤19 years old comprised 69% to 84% annually, with companion shifts between 13% and 24% for ages 20–29 and 3% and 7% for ages 30–77.

The female proportion was fixed (M1) or remained at (M2) 0.85 across age groups annually, which also comprised the annual median female proportion for all remaining models. Predictably, ±5% variability in cohort sex ratio in model M3 introduced a comparable annual range (0.80–0.88) across age groups during the 165‐year run, but with a much smaller inter‐quartile range (IQR), 0.82–0.86, across years. Similarly, ±10% variability in cohort sex ratio in model M4 produced a predictable range (0.75–0.92) with comparable IQR (0.79–0.87), and nearly identical sex ratio proportions across age groups were also observed for model M5.

**FIGURE 1:**
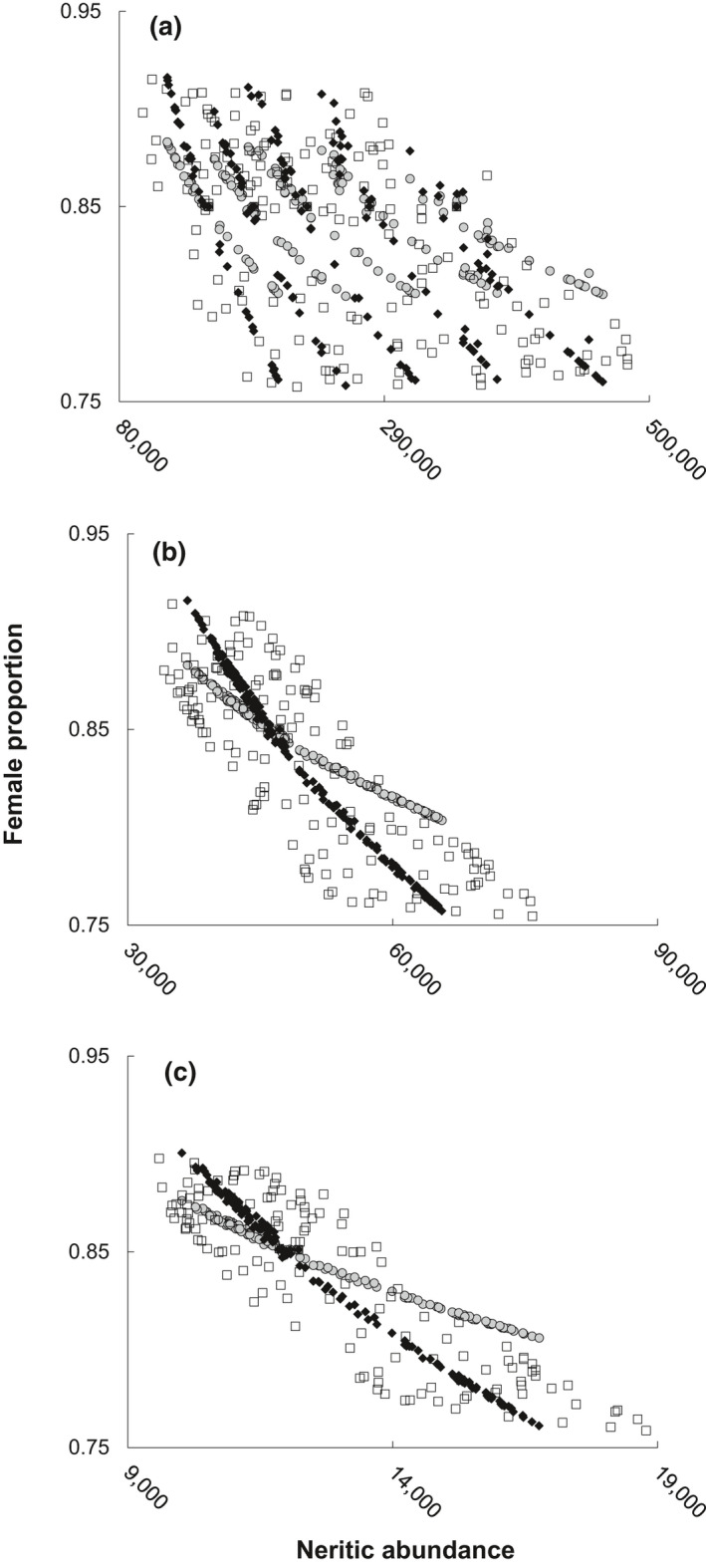
Annual (*n* = 165) female proportion (*y*‐axis) and abundance (*x*‐axis) across model runs with variable cohort sex ratio (M3 = circles, M4 = diamonds, and M5 = squares) for each of three age groups: NR–19 (panel a); 20–29 (panel b); 30–77 (panel c)

Variable but always significant (*p* < 0.001) association strengths were noted across age groups with respect to annual abundance and the corresponding female proportion. Due to high volatility in annual abundance across models for the youngest neritic loggerhead sea turtles (Figure 1a), greatest correlation strength was associated with model M2 (−0.67) with decreasing correlation strength across remaining models: M3 (−0.62), M4 (−0.62), and M5 (−0.59). Stronger correlation strengths were associated with age 20–29 loggerhead sea turtles (Figure 1b), with greatest strengths (−0.99) in models M3 and M4 when NR age and sex ratio varied as a function of the AMO, but less so when only NR age varied (M2 = −0.81) or when cohort abundance was also varied as a function of the AMO (M5 = −0.80). For age 30–77 loggerhead sea turtles (Figure 1c), strongest negative correlation (−1.00) was also associated with models M3 and M4, but with less change relative to ages 20–29 for models M5 (−0.87) and M2 (−0.82).

**FIGURE 2:**
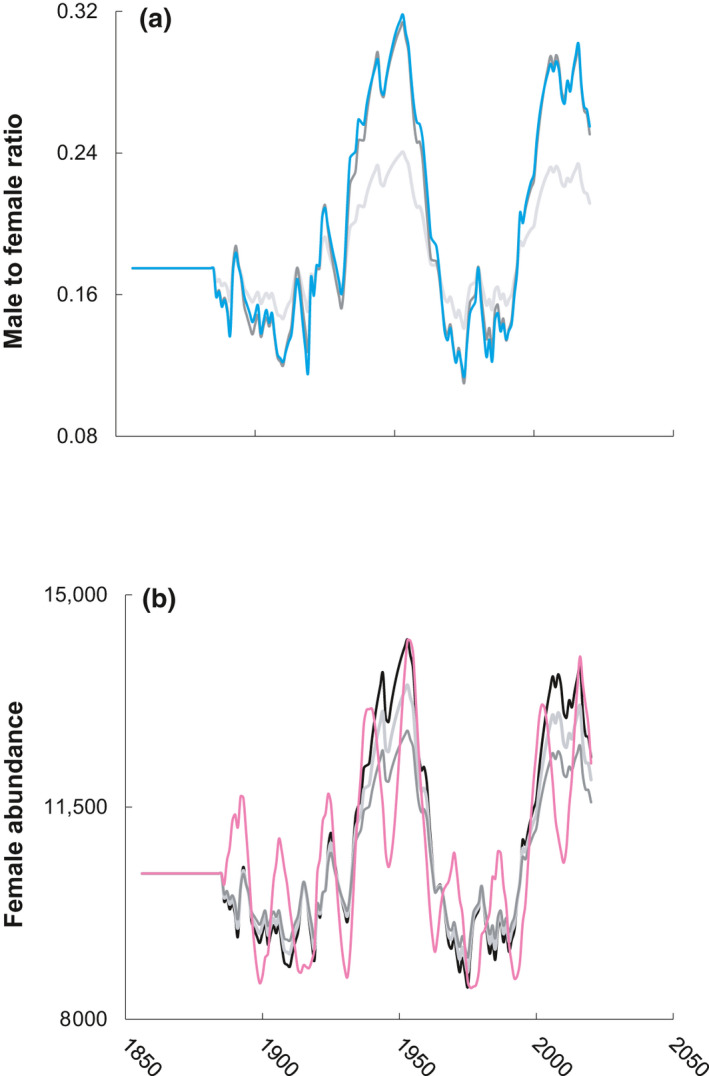
Temporal synchrony in male to female ratio (panel a) and female abundance (panel b) for age 30–77 loggerhead sea turtles across model runs. Across panels, series denote models as follows: M2 (black line, panel b only), M3 (light gray), M4 (dark gray), and M5 (dashed lines)

Within the age 30–77 group, median male to female ratio (0.17) in models M2 to M5 matched M1, with negligible change between models M2 and M1 but with a predictable gradient of annual distribution variation reflecting proportionate sex in models M3 and M5 (Figure 2a). Annual ratio change relative to the median value was highly skewed toward increased vs. decreased magnitude, as evidenced by annual maximum of 0.32 but annual minimum of only 0.11 (Figure 2a). Furthermore, for this age group, significant (*p* < 0.001) positive correlation was observed between peak male to female annual ratio scores and absolute female abundance (Figure 2b) for models M2 (*r* = 0.82), M3 (1.00), M4 (0.99), and M5 (0.77).

### 3.2 Objective 2

T data were analyzed for 2330 loggerhead sea turtles captured by trawling in coastal waters between Winyah Bay, SC and St. Augustine, FL between 2000 and 2019, of which only 113 (5%) were not reliably classified as male or female. Overall, females comprised 0.67 (*n* = 1484) and males 0.33 (*n* = 733) of loggerhead sea turtles that were successfully sexed.

Across all growth rate equations, the estimated age structure of male and female loggerhead sea turtles captured by trawling was significantly different (*χ* ^2^ = 928.637, df = 6, *p* < 0.001) from the base model (M1), also the median distribution among remaining models. Across years, and assuming the reconstructed growth rate equation, loggerhead sea turtles ≤19 years old comprised 56% of captures, ages 20–29 comprised 37%, and ages ≥30 comprised 7% Under the assumption of alternative growth rates, estimated age structure shifted to 39%, 49%, and 13% for 5% slower growth, but 71%, 25%, and 4% for 5% faster growth.

**FIGURE 3:**
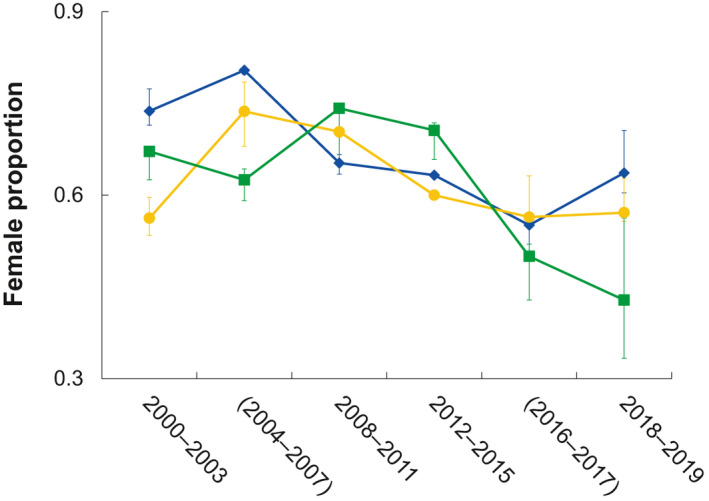
Temporal distribution of the proportion of female loggerhead sea turtles (*y*‐axis) among age group (NR–19 = blue diamonds, 20–29 = yellow circles, and ≥30 = green squares) estimates following capture by research trawling between 2000 and 2019. For all series, error bars denote the maximum and minimum female proportion across growth rate equations. Survey years with parentheses denote trawling in the Charleston, SC shipping channel

Across age groups, variable temporal trends were observed in the female proportion of loggerhead sea turtles captured by research trawling between 2000 and 2019 (Figure 3). Significant temporal decline was associated with loggerhead sea turtles ≤19 years old (*p* = 0.001, *r* = −0.72) and ≥30 years old (*p* = 0.018, *r* = −0.55); however, temporal decline in the latter group was also significantly correlated (*p* = 0.006, *r* = 0.62) with annual sample size, which also significantly declined (*p* = 0.014, *r* = −0.57) across temporal survey blocks. No significant correlations for any size group were associated with growth rate equation used to assign age, consistent with minor female proportion variability across age assignment equations (Figure 3).

## 4 DISCUSSION

Across disciplines, the scientific literature abounds with existential deliberations of the delicate balance between “natural” ecological instability and global anthropogenic ramifications. At the grandest scale, some researchers contend that the breadth of impacts from *Homo sapiens* warrants classifying the last 500 years as the “Anthropocene” epoch (Lewis & Maslin, 2015), while others suggest that per capita landscape transformations several thousand years ago were far more influential on, and in turn detrimental to, the global atmosphere (Ruddiman, 2013). Regardless of causality, climate variability at geological scales validates that species whose sex is determined by ambient conditions during embryonic development must be highly resilient. Extant sea turtle species exemplify this sentiment by persistence across geological bottlenecks that decimated numerous other taxa (Scheyer et al., 2014). However, that impressive history seems to provide little reassurance for future populations, as evidenced by pervasive pessimism in the sea turtle climate literature (Patrício et al., 2021).

Across sea turtle species, sentiments of despair stem largely from myopic emphasis on hatchling sex ratios and projections of future incubation conditions (Hawkes et al., 2009; Patrício et al., 2021), despite incubation interactions which complicate such projections (Wyneken & Lolavar, 2015). Operational sex ratio research comprises a secondary topical theme but is more indicative of breeding phenology than grand population sex ratio (Hays et al., 2014; Lasala et al., 2018). Conspicuously absent, however, from >250 publications collectively reviewed by Hawkes et al. (2009) and Patrício et al. (2021) were investigations of cross‐sectional cohort sex ratios on foraging grounds using circulating blood testosterone concentration (Owens et al., 1978). Such studies have occurred globally across sea turtle species, but most extensively in the NWA, the Gulf of Mexico, and the Caribbean Sea. Sex ratio data for loggerhead sea turtles in the NWA span four decades and eight degrees of latitude (Arendt, Boynton, Schwenter, Segars, Byrd, Whitaker, Parker, Owens, et al., 2012; Arendt, Schwenter, et al., 2012; Braun‐McNeill et al., 2007, 2016; Wibbels et al., 1991). Less extensive regional data collectively chronicle green (*Chelonia mydas* [Linnaeus, 1758]; Bolten et al., 1992; Bagley, 2003; Sanchez, 2013), hawksbill (*Eretmochelys imbricata* [Linnaeus, 1766]; Diez & van Dam, 2003; Blanvillain, Wood, et al., 2008; Hawkes et al., 2013; Gorham et al., 2014), and Kemp's ridley (*Lepidochelys kempii* [Garmin, 1880]; Gregory & Schmid, 2001; Geis et al., 2005; Witzell et al., 2005) sex ratios. Across studies, far fewer females were encountered on foraging grounds than represented as hatchlings in natal rookeries, suggesting broad‐scale demographic regulation.

With few exceptions, ontogenetic development of sea turtle species spans the geographic extent of oceanographic gyres associated with respective basins; thus, the importance of large‐scale currents on subsequent oceanic distribution and foraging success is widely recognized (Lambardi et al., 2008; Polovina et al., 2006). Active swimming against currents as young juveniles is also demonstrated across species but appears to provide a secondary versus primary role in transport and distribution (Briscoe et al., 2016; Putman & Mansfield, 2015). As such, oceanic currents should greatly structure cohort recruitment via variable foraging opportunities (Ascani et al., 2016).

Although compensatory growth (Bjorndal et al., 2003) may minimize variability in oceanic stage duration (Avens et al., 2013), as demonstrated herein, substantial cohort resonance can occur due to seemingly subtle differences in NR age. Cohort resonance is further exacerbated when departing lower survival habitats at a younger age as is presumed for oceanic vs. neritic loggerhead sea turtles across populations (Conant et al., 2009). However, as modeled herein, appreciable shifts in neritic sex ratio were only achieved when shifts in abundance were autocorrelated with variable cohort sex ratio, and sole manipulation of the latter also does not achieve appreciable temporal change in female abundance (Arendt, 2016).

With no annual deviation in stage‐based survival rates, our models produced substantial fluctuation in neritic assemblage abundance and sex ratio, but critically, the modeled population remained stable via a companion inverse oscillation in the oceanic realm. As such, we caution that this observation cannot be disregarded when considering contemporary variability in sea turtle populations, which from a geological perspective should predominantly reflect long‐term, climate‐mediated, stable oscillations. This suggestion is consistent with a review by Leigh (1981) that redistribution of taxa relative to the status quo merely serves as a harbinger of changing climate, but devoid of necessity that change be implicitly good or bad. Without appreciation for the long‐term resilience of populations to expand and contract in both distribution and habitat‐centric demography, it is understandable why such change would be worrisome under unidirectional forecasts (Chaloupka et al., 2008). Therefore, the model results presented herein as well as elsewhere under variable annual survival (Arendt, M., & Schwenter, J., in review) provide an alternative perspective. Furthermore, the long periodicity of the AMO and cycle congruence (Nye et al., 2014) during both pre‐ and post‐petroleum‐based economies should also instill at least a modicum of encouragement.

A second paradigm shift that we wish to convey is the discouragement of univariate thinking. Cohort shaping does occur early in life, particularly for long‐lived species with inherently high annual survival requirements (Ascani et al., 2016; Halley et al., 2018). However, assemblages reflect a tapestry of cohorts, with individual cohorts, particularly at the adult stage, comprising a small fraction of the overall assemblage (Arendt et al., 2013). As such, resonant effects require grand gestures, such as harmonious return of all cohort members to the neritic realm at a specified age, as modeled herein. Although overly simplistic, this assumption was included to demonstrate the magnitude of variability needed across cohorts to produce such change. Furthermore, stable survival trajectories for long‐lived species consist of low survival early in life but much higher annual survival in later stages (Conant et al., 2009); thus, making a low number even lower (or conversely, slightly higher) exerts considerably less net assemblage resonance than when groups of cohorts align in clusters that are distinguished across decades. Lastly, in the absence of stable cohort cluster parsing, populations are more likely to crash; thus, annual survival alone, particularly at later life stages, represents the least likely consideration.

Lack of temporally increased female skew in the coastal trawl survey conflicts with such suggestion for a globally important foraging ground for green sea turtles (Jensen et al., 2018). Explanations for differing results include a longer time series in the present study and therefore greater cross‐sectional sampling of cohorts, as well as greater spatial origin of sampled cohorts.

Among the seven extant sea turtle species, loggerhead sea turtles exhibit the greatest latitudinal range in nesting, particularly in the northern hemisphere (Wallace et al., 2010), which should diversify annual cohort sex ratios. However, because NWA loggerhead nesting at lower latitudes comprises ~90% of annual hatchling production (NMFS & USFWS, 2008), annual cohorts are mostly female (Mrosovsky & Provancha, 1992; Wyneken & Lolavar, 2015). Predictably, females remain prevalent across oceanic (Delgado et al., 2010) and neritic foraging grounds (Arendt, Boynton, Schwenter, Segars, Byrd, Whitaker, Parker, Owens, et al., 2012; Arendt, Schwenter, Segars, et al., 2012; Wibbels et al., 1991). Consistent with trends for young juveniles in oceanic habitats preceding NR into our survey area (Delgado et al., 2010), we also observed modest temporal oscillation in the female proportion in the NR to age 19 group. However, modest variability in female prevalence, particularly in later survey years, may have also been amplified through reduced annual sample size, particularly given an ideal asymptotic threshold of 100 to 140 samples per analytical block (Shertzer et al., 2018).

In addition to not supporting the onset of population transition toward becoming irreversibly male limited (Hawkes et al., 2007), AMO‐mediated NR age consistently synchronized peak adult male and adult female abundance across decades. Support for the model assumption of reduced female proportion during the AMO cold phase comes from the analysis of 100 years of reconstructed soil temperatures at nine distinct areas encompassing 95% of annual nesting for NWA loggerhead sea turtles (Arendt, 2016). While the resolution of these data was not sufficient for predicting sex ratio, generalized treatment of temporal variability in our models revealed a logical but hitherto unreported contribution to breeding phenology and operational sex ratio. Peak synchrony of adult males could also not occur without increasing male prevalence for several decades prior, which provides a more optimistic alternative explanation for reduced female abundance among trawl survey captures than reduced female hatchling fitness under warmer incubation conditions (Fisher et al., 2014; Laloë et al., 2017). Likewise, it should not be forgotten that modeled scenarios that produced peak synchrony also contained no variability in annual stage‐based survival rates, but still generated dramatic shifts in the relative abundance of NR to age 19 loggerhead sea turtles, lending even further support to the notion that worst‐case scenario does not warrant de facto interpretation.

The greatest mystery of temporal variability in loggerhead sea turtle sex distribution remains the 20% disparity between the proportion of female hatchlings entering the ocean annually and their relative occurrence reported across multiple foraging grounds. Spatial concentration of annual nesting of NWA loggerhead sea turtles at lower latitudes produces some of the most female‐biased sea turtle nests in the world (Mrosovsky, 1994). Consequently, annual variability in the proportion of females in this region would need to vary by at least 20% to achieve the relatively stable female proportion reported across time and space, at least in the southeast United States (Arendt, Boynton, Schwenter, Segars, Byrd, Whitaker, Parker, Owens, et al., 2012; Braun‐McNeill et al., 2007; Shoop et al., 1998; Wibbels et al., 1991). Annual variability in the proportion of female hatchlings is reported for traditionally strongly female‐biased beaches in Florida (Wyneken & Lolavar, 2015), but not at the magnitude and frequency needed to mathematically achieve the sustained reduction in female distribution associated with foraging grounds. As such, acute differential mortality of female hatchlings may be the root of this discrepancy (Carthy et al., 2003), given the greatest sex ratio discrepancy between hatchling and oceanic stages (Delgado et al., 2010).

Rather than continued perception of climate variability as an existential threat to species with ESD, we suggest reconsidering climate to be an environmental component of density‐dependent regulation. Across neritic age groups, modeling herein demonstrated strong inverse relationships between the proportion of females and the abundance of the respective age group. Reciprocally, this observation in turn conveys that well‐sampled sex ratios must therefore be abundance proxies. Greatest variability in abundance and least reliable association between sex ratio and abundance was associated with the youngest neritic individuals, further evidence of the magnitude of fluctuation that younger age groups can withstand while oscillating along a stable trajectory. Therefore, assuming acute loss of female hatchlings as the predominant driver of 20% fewer foraging females than hatchling females is not unto itself problematic if viewed in the context of density‐dependent population regulation. While contemporary climate tends toward the warm end of the spectrum experienced during the past century, it is not unprecedented either (Viau et al., 2002). As such, future assessments of sex ratio in the coming decades represent an exciting opportunity to observe if the patterns herein persist. Likewise, our findings highlight the importance of concurrent monitoring of size/age structure, relative abundance, and sex ratio to be certain that future change is aligned with model expectations and not misinterpreted as an unsustainable phenomenon (Monsinjon et al., 2019).

## CONFLICT OF INTEREST

No conflict of interest.

## AUTHOR CONTRIBUTIONS

MDA managed data collection as the permit and grant PI, supervised animal capture and handling as chief scientist for research cruises, completed all statistical analyses, and compiled the manuscript. JAS supervised animal capture and handling as chief scientist for research cruises. JAS completed testosterone RIA from 2010 through 2018, and which were conducted in the laboratory of and in consultation with DWO. RAV conducted an ELISA in 2019. All co‐authors also provided editorial contributions.

## Data Availability

Upon request to the senior author, matrix model outputs (objective 1) and sex assignments (objective 2) can be made available to verify the accuracy of the findings reported herein.
